# Gas-Phase Hydration Thermochemistry of Sodiated and Potassiated Nucleic Acid Bases

**DOI:** 10.1007/s13361-012-0436-5

**Published:** 2012-07-21

**Authors:** Henryk Wincel

**Affiliations:** Institute of Physical Chemistry, Polish Academy of Sciences, 01-224 Warsaw, Poland

**Keywords:** Hydration energies, Cationized nucleobases, High-pressure mass spectrometry

## Abstract

Hydration reactions of sodiated and potassiated nucleic acid bases (uracil, thymine, cytosine, and adenine) produced by electrospray have been studied in a gas phase using the pulsed ion-beam high-pressure mass spectrometer. The thermochemical properties, *ΔH* ^*o*^_*n*_, *ΔS* ^*o*^_*n*_, and *ΔG* ^*o*^_*n*_, for the hydrated systems were obtained from hydration equilibrium measurement. The structural aspects of the hydrated complexes are discussed in conjunction with available literature data. The correlation between water binding energies in the hydrated complexes and the corresponding metal ion affinities of nucleobases suggests that a significant (if not dominant) amount of the canonical structure of cytosine undergoes tautomerization during electrospray ionization, and the thermochemical values for cationized cytosine probably correspond to a mixture of tautomeric complexes.

## Introduction

DNA bases represent the most important biomolecules which, among other functions, store and transmit genetic information. The structure and biological function of DNA is strongly influenced by interactions of its components with aqueous surroundings [1–3]. The interaction of DNA with the environment has been the subject of many investigations using X-ray crystallography, NMR, femtosecond spectroscopy and computational methods [4–15]. Water and metal ions are essential for DNA’s stability and function. The important role of water and the participation of counter ions in the modulation of the three-dimensional structure of DNA have been much discussed in the literature [1, 4–6, 9–15]. While the destabilizing repulsion between negatively charged phosphate groups of the bases is neutralized by metal cations, hydration is crucial for the stability of the duplex of DNA [1, 9, 10]. Water can also affect the tautomeric equilibrium between the canonical structures of nucleobases and their “rare” tautomeric forms [16–34] that differ from the canonical structures in the Watson-Crick pairing scheme of DNA, and may be implicated in spontaneous mutations [23, 24, 35–40].

The principal monovalent cations in living systems are Na^+^ and K^+^, which are not only involved in DNA compaction but also play an important role in the regulation of other biological processes, such as homeostasis and cellular function. Molecular dynamics (MD) simulations show that the distribution of these ions around DNA is different: Na^+^ binds predominantly to the oxygen atoms of the phosphate group, while K^+^ ions interact with the electronegative sites of the DNA bases in the major and in the minor groove [9, 41]. The Na^+^ ion plays a primary role in maintaining the compact structure of DNA in vivo and making the water structure around DNA more organized and less mobile compared with K^+^ [42]. Water and ions form more stable long-lived pairs with the sites on DNA bases than near the phosphate groups [9]. This has a clear implication for conformational recognition of DNA through the study of the interactions of nucleobases with metal ions and water molecules. In this context, numerous studies have been carried out in the gas phase to investigate both hydration and metal ion interactions with individual nucleobases to obtain new insight into how solvent and electrostatic interactions influence the DNA base structure.

A number of techniques, including tandem mass spectrometry [43–46], guided ion beam experiments [47–51], IRMPD spectroscopy [52, 62–64], and theoretical calculations [24, 47–64] have been used to determine the binding of metal ions to nucleobases, the relative populations of tautomeric forms of these complexes, and their tautomerization barriers. Structural information about cationized nucleobases such as uracil, thymine, cytosine, adenine, and quinine, and the binding strength of metal ions in these systems has been inferred from various collision-induced dissociations [43, 47–51] and theory [46–51, 55–59]. In studies by Rodgers and coworkers [52], IRMPD spectra and calculations provided detailed information about the gas phase structures of sodium cationized uracil and five thiouracils generated by electrospray. Similarly, Fridgen and co-workers [62–64] used IRMPD spectroscopy in combination with computational methods to determine the structures of hydrated Ura_n = 1,2·_–Li^+^ and Thy_n = 1,2_–Li^+^ [62], (Ade-Thy)–Li^+^ [63], and Ade–M^+^, M = Li, Na, K, and Cs [64] complexes.

Herein, we report the first experimental results on the water binding energies to cationized nucleic acid bases (NABs), Ura–M^+^, Thy–M^+^, Cyt–M^+^, and Ade–M^+^, where M = Na or K obtained from gas-phase equilibrium determination.

## Experimental

The gas-phase hydration experiments were performed with a home-made 60° magnetic sector high-pressure mass spectrometer (HPMS) using a pulsed ion-beam ESI ion source, which has been previously described in detail [65]. Briefly, cationized NABs were obtained by electrospray from a silica capillary (15 μm i.d., 150 μm o.d.) The solution containing ~2.0 mM NAB in water/methanol (1:1) mixture and NaCl or KI was supplied to the capillary by a syringe pump at a rate of 0.8 μL/min. The samples studied were purchased from Aldrich Chemical Co. (Steinheim, Germany) The clustered ions were desolvated by a dry nitrogen gas counter-current and in a heated (~80 °C) pressure-reducing capillary through which they were introduced into the fore-chamber, and then deflected toward a 3-mm orifice in the interface plate leading to the reaction chamber (RC) Ions drifting across the RC toward the exit slit under the influence of a weak electric field (2 V/cm at 10 mbar) were hydrated and reached equilibrium prior to being sampled to the mass analysis section of the mass spectrometer. Ion detection was provided by a secondary electron scintillation detector of the Daly type with an aluminum conversion dynode using a short rise-time photomultiplier (Type R-647-04, Hamamatsu Photonics Deutschland GmbH, Germany) The output pulses of the multiplier were counted using a multichannel scaler with dwell-time per channel of 1 μs.

**Figure 1:**
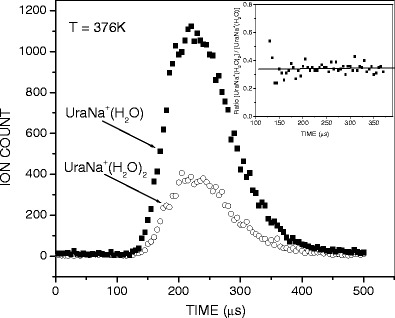
Arrival time distributions of the reactant, UraNa^+^·(H_2_O), and product, UraNa^+^·(H_2_O)_2_, ions. The inset shows the ratio of ion intensities, [UraNa^+^·(H_2_O)_2_]/[UraNa^+^·(H_2_O)], as a function of ion residence time

Mass spectra were registered with continuous ion sampling, while for equilibrium determination the ion beam was injected into the RC in a pulsed mode by applying short pulses (+50 V, 90 μs) to the deflection electrode with repetition of 1 ms. Typically, several thousand injection pulses were sufficient to accumulate a reasonable signal of the ion arrival time distribution (ATD) for each mass on the multichannel scaler (Figure 1).

The reagent gas mixture consisting of pure N_2_ as the carrier gas at about 10 mbar and a known partial pressure of water vapor (0.02–0.20 mbar) was supplied to the RC via the heated reactant gas inlet (RGI) at a flow rate of ~100 mL/min. The pressure was measured with an MKS capacitance manometer attached near the inlet of the RGI. The amount of water introduced into the N_2_ gas flow was kept constant throughout the temperature-dependent measurements of the equilibrium constants. Water concentrations were controlled continuously with a calibrated temperature and humidity transmitter (Delta OHM, Type DO 9861T; Casselle di Selvazzano, Italy) inserted into the carrier gas flow line. The RC temperature was monitored by an iron-constant thermocouple, which was embedded close to the ion exit slit; the temperature could be varied from ambient to 300 °C by electrical heaters.

## Results

**Figure 2:**
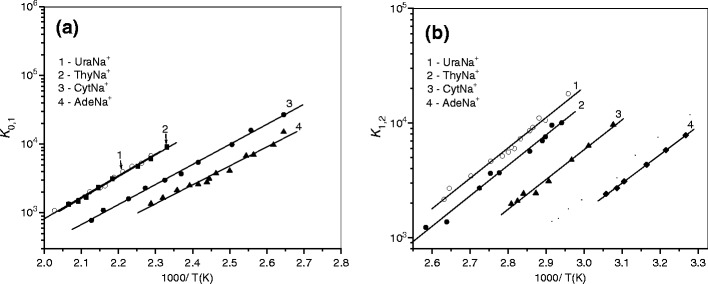
van’t Hoff plots of equilibrium constants for the gas-phase reactions NAB-Na^+^·(H_2_O)_*n*-1_ + H_2_O < == > NAB-Na^+^·(H_2_O)_*n*_: **(a)** *n =* 1, and **(b)** *n =* 2

**Figure 3:**
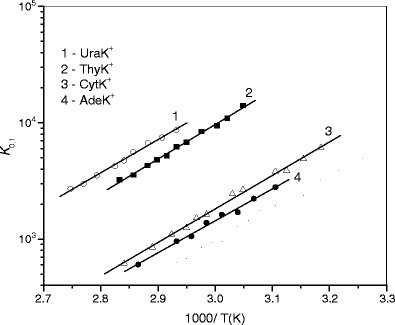
van’Hoff plots of equilibrium constants for the gas-phase reactions NAB-K^+^ + H_2_O < = > NAB-K^+^·(H_2_O)

In the present work, the thermochemical properties for gas-phase clustering reactions (1)

1 $$ {\text{NAB}} - {{\text{M}}^{ + }}\cdot {\left( {{{\text{H}}_{{2}}}{\text{O}}} \right)_{{n - }}}_{{1}} + {{\text{H}}_{{2}}}{\text{O}} \leftrightarrow {\text{NAB}} - {{\text{M}}^{ + }}\cdot {\left( {{{\text{H}}_{{2}}}{\text{O}}} \right)_n} $$

were obtained from temperature-dependent measurements of the equilibrium constants, *K*_*n-*1,*n*_, Equation (2)

2 $$ {K_{{n - }}}_{{{1},n}} = \left( {{{{{{{I_n} \cdot {P_o}}} \left/ {I} \right.}}_{{n - 1}}} \cdot P} \right) $$

where *I*_*n*_ and *I*_*n-*1_ are recorded ATD peak areas of NAB-M^+^·(H_2_O )_*n*_ and NAB-M^+^·(H_2_O )_*n-*1_, respectively, and *P* is the known partial pressure of water (in mbar) The standard pressure *P*_*o*_ is 1000 mbar. Equilibrium attainment in the RC was verified by comparing the ATDs of the reactant and product ions, and testing that the *I*_*n*_ */I*_*n-*1_ ratio is independent of ion residence time. A typical example of such tests is shown in Figure 1 for the (1, 2) hydration step of UraNa^+^. The inset of the figure shows that within the error limits and the limits of statistical noise, the ratio [Ura-Na^+^(H_2_O)_2_]/[Ura-Na^+^(H_2_O)] remains essentially constant, suggesting the attainment of equilibrium for the system. Figures 2 and 3 give the van’t Hoff plots for the equilibria 1. The standard enthalpy, *ΔH* ^*o*^_*n*_, and entropy, *ΔS* ^*o*^_*n*_ , values for reactions 1 obtained from the van’t Hoff plots are shown in Tables 1 and 2, together with the free energy, *ΔG* ^*o*^_*n*_, values obtained from $\Delta {G^o}_n = \Delta {H^o}_n--T\Delta {S^o}_n$. The weighted least-squares fitting procedure was used to obtain the slopes and intercepts of each line. Only data for small *n* are given in the tables, because determining of the enthalpies for higher hydration steps requires equilibration temperatures below room temperature, which is not accessible with the present reaction chamber.

**Table 1:** Experimental enthalpies, entropies and free energy values^a^ for the hydration of the sodiated nucleic acid bases

Ion	*n*	*–ΔH* ^*o*^_*n*_	*–ΔS* ^*o*^_*n*_ *–*	*ΔG* ^*o*^_*n*_	MIA
		(kJ/mol)	(J/mol K)	(kJ/mol)^b^	(kJ/mol)
Ura-Na^+^	1	62.3(2)	66.5(4)	42.6(3)	145.2 (**1a**)^c^
	2	50.6(2)	70.3(5)	29.7(3)	
Thy-Na^+^	1	61.1(2)	66.1(7)	41.4(4)	145.6 (**1a)** ^c^
	2	51.0(2)	73.2(6)	29.0(4)	
Cyt-Na^+^	1	57.7(2)	69.0(5)	37.0(5)	177^d^
	2	50.2(3)	77.8(7)	27.0(5)	
Ade-Na^+^	1	53.6(3)	62.0 (6)	35.1(5)	203.8 (**3c**)^c^
	2	47.7(1)	83.7(3)	22.8(2)	
Na^+^	4	52.7(2)	100.4(8)	22.8(4)	
		52.7^e^	99.2^e^	23.0^e^	
		53.1^f^	92.0^f^	25.5^f^	

Standard pressure is 1000 mbar

^a^Uncertainties in parentheses

^b^ *–ΔG* ^*o*^_*n*_ at 298 K

^c^Calculated values from Reference [56]

^d^Weighted value using MIA’s (kJ/mol) from Reference [56]: 212.5 (**2d**), 165.7 (**2e**), 179.0 (**2f**), and 137.7 (**2g**), and mole fractions of cytosine: 0.22(**2**), 0.44(**2a)**, 0.26(**2b**), and 0.08(**2g**) from Reference [68], see text

^e^Reference [66]

^f^Reference [67]

**Table 2:** Experimental enthalpies, entropies, and free energy values^a^ for the hydration of the potassiated nucleic acid bases

Ion	*-*Δ*H* ^*o*^ *n*	*-*Δ*S* ^*o*^ *n*	*-*Δ*G* ^*o*^ *n*	MIA
	(kJ/mol	(J/mol K)	(kJ/mol)^b^	(kJ/mol)
Ura-K^+^	57.3(2)	93.3(3)	29.5(3)	108.4 (**1a**)^c^
Thy-K^+^	56.9(1)	94.6(4)	28.8(2)	107.1 (**1a**)^c^
Cyt-K^+^	55.6(1)	103.8(3)	24.7(2)	126^d^
Ade-K^+^	52.7(3)	97.9(4)	23.5(4)	154.4 (**3c**)^c^

Standard pressure is 1000 mbar

^a^Uncertainties in parentheses

^b^ *–ΔG* ^*o*^_*n*_ at 298 K

^c^Calculated values from Reference [56]

^d^Weighted value using MIAs (kJ/mol) from Reference [56]: 159.0 (**2d**), 111.3 (**2e**), 132.6 (**2f**), and 94.6 (**2g**), and mole fractions of cytosine: 0.22(**2**), 0.44(**2a)**, 0.26(**2b**), and 0.08(**2g**) from Reference [68], see text

We determined the thermochemical data for the reaction 3 to support the validity of the present results and provide a bases for comparison with the data obtained by high-pressure mass spectrometry in other laboratories.

3 $$ {\text{N}}{{\text{a}}^{ + }} \cdot {\left( {{{\text{H}}_{{2}}}{\text{O}}} \right)_{{3}}} + {{\text{H}}_{{2}}}{\text{O}} \leftrightarrow {\text{N}}{{\text{a}}^{ + }} \cdot {\left( {{{\text{H}}_{{2}}}{\text{O}}} \right)_{{4}}} $$

Table 1 shows that the present values are in good agreement with the reported data [66, 67].

## Discussion

### Uracil and Thymine

**Scheme 1:**
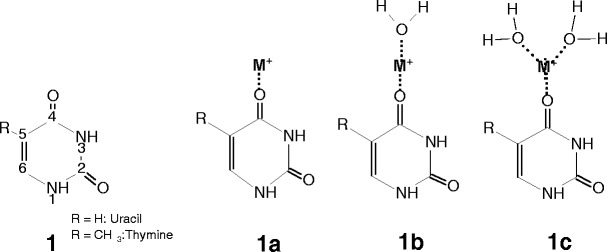
Structures of the uracil and thymine and their complexes with a metal ion M^+^ (M^+^ = Na^+^ and K^+^) and water molecule

Uracil and thymine can exist in various tautomeric forms, differing in the position of the protons which may be bound to either nitrogen or oxygen atoms. Theoretical studies [56] indicate that the canonical keto form, **1**, is at least 45 kJ/mol more stable than other possible structures of these two bases. Similarly, among the tautomers of the Ura–M^+^ and Thy–M^+^ complexes, the most stable structure is predicted [47, 56, 58] to be **1a**, in which the metal ion interacts with the O4 atom of the **1** tautomer. The metal binding to uracil and thymine at O2 atom of **1** was found [47] to be higher in energy by 11.7 and 5.0 kJ/mol for Na^+^, and 11.6 and 4.8 kJ/mol for K^+^, respectively. Also, the bicoordinated binding to the non-canonical tautomers, N3···M^+^···O2 and N3···M^+^···O4, was calculated [56] to be less favorable than that of **1a** (Scheme 1).

Thus, it might be expected that the ground-state **1a** tautomer of both Ura-M^+^ and Thy-M^+^ systems should be the dominant species in the gas phase experiments. However, the question arises whether this structure is formed from the solution by electrospray ionization? The IRMPD action spectra of Ura-Na^+^ in the region ~1000–1900 cm^–1^ measured by Nei et al. [52] and their calculations of linear IR spectra for Ura-M^+^ (M = Li, Na, K, Rb and Cs), indicate that **1a** is the dominant species generated by electrospray. Also, Fridgen and co-workers [62] in their IRMPD spectroscopy study between 2500 and 4000 cm^–1^ and calculations clearly show that the Li^+^ cation in the Ura–Li^+^ and Thy–Li^+^ complexes produced by electrospray is coordinated to the O4 atom, and the first two water molecules in each of these complexes are attached to Li^+^. Therefore, it is reasonable to expect that the metal ion coordination in the Ura–M^+^ and Thy–M^+^ complexes generated in our experiments by electrospray and water binding in these complexes should be similar to those determined in the studies [52, 62]. Thus, one may assume that the **1a** complex will be the dominant ionic precursor for the **1b** and **1c** structures involved in the hydration equilibrium measurements 1**.** The water binding energies in these structures are given in Tables 1 and 2.

### Cytosine

**Scheme 2:**
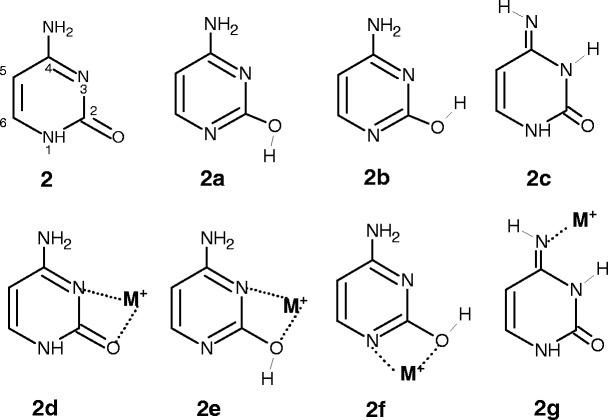
Structures of the cytosine tautomers and their complexes with a metal ion M^+^ (M^+^ = Na^+^ and K^+^) considered in this study

Many studies have been conducted on the tautomeric forms of cytosine and their relative stabilities [17–20, 22–24, 30, 35, 51, 55–58, 68, 68]. The four lowest-energy tautomers, **2–2c,** lying within the range of ~13 kJ/mol, and the corresponding complexes with metal ions, **2d–2g** [51, 56, 58], are shown in Scheme 2.

For the gas phase, most of quantum chemistry results [35, 68, 68] predict that the enol form, **2a**, is the lowest energy tautomer, and **2b**, **2**, and **2c** lie at 2.9–3.1, 3.2–6.9, and 4.8–13.4 kJ/mol above **2a**, respectively**.** In the case of the **2d**, **2e**, **2f**, and **2g** complexes**,** the calculated [56] relative energies are: 0.0, 59.8, 41.0, and 83.3 kJ/mol for Cyt-Na^+^, and 0.0, 54.0, 36.8, and 75.3 kJ/mol for Cyt–K^+^, respectively. Very recently, Bazsó et al. [68] reported the relative population ratios of the **2**, **2a**, **2b,** and **2c** tautomers as 0.50, 1.00, 0.59, and 0.18, respectively, produced by thermal evaporation at ~450 K and ~430 K for IR and UV spectroscopic measurements, respectively. These populations, however, can be related to the vapor state. In the context of the present experiments, one might ask about the tautomer populations in an aqueous solution? Theoretical and experimental studies [17] show that the canonical N1-H aminooxo tautomer, **2**, predominates in water, and the free energy of tautomerization of this structure into any other forms is predicted to be greater than 24 kJ/mol [35]. Spectroscopic measurements [17] indicate that in an aqueous solution, **2** very slightly (~3 × 10^-3^) converts into the N3–H aminooxo form. This may suggest that **2** should lead predominantly to the **2d** complex under electrospray ionization. However, as will be discussed below, the correlation between the water binding energies to NAB–M^+^ and the metal ion affinities (MIAs) of NABs implies that one or more tautomers having MIA lower than that of **2d** contribute substantially to the populations of the Cyt-M^+^ complexes. The energy barriers calculated [51] at 0 K for the **2d → 2e** and **2d → 2g** conversions as 174 and 207 kJ/mol for Cyt–Na^+^, and 166 and 200 kJ/mol for Cyt–K^+^, respectively, are too high to be overcome at thermal energies in our experiments. In contrast to the low pressure TCID experiments [51], in the present study the internal energy released during the metal ion–cytosine association is expected to be efficiently thermalized within the electrospray microdroplets and in the atmospheric pressure region. Therefore, we assume that **2** is converted into other forms, **2 → 2a** [20] and **2 → 2c** [23, 30], by water-assisted tautomerization during the final stages of the electrospray droplet lifetime. The calculated barrier heights for the **2 → 2a** and **2 → 2c** processes in monohydrated cytosine are 58 and 20 kJ/mol, respectively, compared with 157 and 44 kJ/mol for isolated systems [20, 23]. The microsolvation results [35] predict some coexistence of the **2**, **2a**, and **2b** tautomers in a microhydrated environment. If this occurs, these tautomers might be the precursors of the corresponding tautomeric complexes, **2d**, **2e**, and **2f**, respectively, for Cyt–M^+^, and their hydrated clusters, Cyt–M^+^·(H_2_O)

### Adenine

**Scheme 3:**
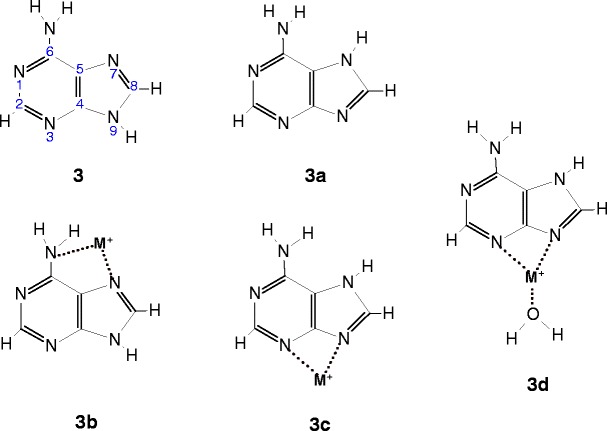
Adenine tautomers and their complexes with a metal ion M^+^ (M^+^ = Na^+^ and K^+^) and water molecule considered in this study

Adenine is one of the most important constituents involved in base pairing with thymine in DNA and with uracil in RNA. There are numerous computational and experimental studies on the tautomers of adenine [16, 21, 24, 26, 28, 31–33, 43, 46–48, 56–58, 60, 61, 64]. In the gas phase, the canonical form **3** is the most stable and predominant species, while the energy of **3a** is higher by ~34 kJ/mol than **3** [46, 56, 61], Scheme 3.

In aqueous solution, the energy difference between **3** and **3a** comes down to 4.7 kJ/mol and the relative Gibbs energy decreases from 38 to 5.2 kJ/mol [61]. T-jump experiments [16], NMR [69] and femtosecond [70] measurements, and calculations [71, 72] show that only the **3** and **3a** tautomeric forms exist in an aqueous solutions, and their population ratio, **3**/**3a**, was estimated to be in the range of 3.6–5.0 at 293 K. Raman spectroscopic results [72] provide evidence for the presence of **3** and **3a**, along with the protonated form of **3** as the predominant species found in aqueous solution at pH = 3. Thus, if adenine complexes with Na^+^ and K^+^ are generated from solution by electrospray, one can expect that the **3** and **3a** tautomers may be involved in the formation of the corresponding complexes, **3b** and **3c**, respectively. However, the IRMPD spectra obtained by Fridgen and co-workers and calculations [64] indicate that the structures of both the Ade–Na^+^ and Ade–K^+^ systems generated by electrospray are similar to that of **3c.** This observation can be attributed [64] to the significant difference between the dipole moments of the **3** and **3a** tautomers in an aqueous solution, which was calculated [72] to be 3.62 and 11.45 D, respectively. Such a situation favors the interaction of **3a** with metal ions to yield **3c,** which in the gas phase is ~43 kJ/mol more stable than **3b** for both Na^+^ and K^+^ [56]. It is very likely that the Ade–M^+^ complexes formed by electrospray in the present experiments and involved in the hydration equilibrium 1 have predominantly a bidentate structure, **3c,** and their singly hydrated species like **3d** [64].

### Correlation Between Water Binding Energies and Metal Ion Affinities

**Figure 4:**
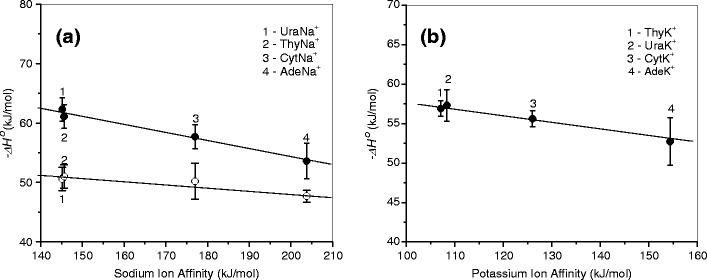
Plot of the binding energies, –*ΔH* ^*o*^_*n*_ at 298 K, for the first (solid symbols) and second (open symbols) water molecule the complexes: **(a)** NAB-Na^+^·(H_2_O)_*n=*1,2_ and **(b)** NAB-K^+^·(H_2_O), versus corresponding metal ion affinity of nucleobases. The metal ion affinity values for Ura, Thy, and Ade are taken from reference [56]. For Cyt, weighted values, see text and Tables 1 and 2

Previous studies [47, 51, 57] have reported that the interactions between cations, Na^+^ and K^+^, and nucleobases, such as Ura, Thy, Cyt, and Ade, in NAB–M^+^ complexes are largely electrostatic in nature. The Mulliken charges retained on the metal ions in these complexes are predicted [47, 51] to be in the range of 0.86–0.97e for NAB–Na^+^ and 0.92–0.99e for NAB–K^+^, depending on the NAB structure. The data also demonstrate that the stronger the binding, the greater the charge being transferred between nucleobases and metal ions during complexation. The positive charge located on the metal ion in the NAB-M^+^ system is expected to depend on the electron withdrawing effect of NAB, determining the magnitude of the MIA value to NAB. The amount of charge on the metal ion in NAB–M^+^ should decrease as the MIA of NAB increases. This order can also be related to the binding strength between NAB–M^+^ and H_2_O in the NAB–M^+^·(H_2_O) systems, where water binds directly to the metal ion through oxygen [62, 64]. Such a correlation has been observed in our previous studies for the binding energies of water to cationized amino acids and monosaccharides [73–75]. As discussed above, the **1a** structure of Ura–M^+^ and Thy–M^+^, and **3c** of Ade–M^+^ solely or as the predominant tautomeric complexes, corresponds to the precursor ions for hydration in the present experiments. In the case of Cyt–M^+^, it is difficult to obtain meaningful data on the population of the tautomers to be involved in hydration equilibrium 1. If Cyt–M^+^ represents a mixture of tautomeric forms, then the MIA for the system will be the sum of the MIA contributions of the particular tautomers weighted by their population. Using the same mole fractions for **2d, 2e, 2f** and **2g** as those of their precursors, **2** (0.22), **2a** (0.44), **2b** (0.26), and **2c** (0.08), respectively, obtained [68] from the matrix isolation IR and UV spectra, and taking the MIA values (Tables 1 and 2) calculated by Russo et al. [56], the weighted MIA values are estimated to be 177 kJ/mol for Cyt–Na^+^ and 126 kJ/mol for Cyt–K^+^. These values are used in Figure 4 showing the plot of the water binding energies, -*ΔH* ^*o*^_*n*=1,2_ , in NAB–M^+^·(H_2_O) versus the corresponding MIAs of NABs. As is evident from the figure, a fair linear correlation exists between these values. When the data is modeled assuming that only the **2d** tautomer is present in our hydration equilibrium measurements, and taking the MIA value of 212.5 kJ/mol [56] or 202.4 kJ/mol [51] for Cyt–Na^+^, and 159 kJ/mol [56] or 160.8 kJ/mol [51] for Cyt–K^+^, the water binding in Cyt-M^+^·(H_2_O) deviates from this trend. It should be noted that the weighted MIA value for Cyt–Na^+^ is in excellent agreement with the experimental value of 177 ± 4 kJ/mol obtained by Cerda and Wesdemiotis [43], where the ions were generated from the condensed phase (under fast atom bombardment), and by Yang and Rodgers [51], who formed their Cyt–M^+^ species in the gas phase directly by interacting the metal ion with a neutral cytosine populated upon thermal evaporation. Moreover, this value is close to the MIA values computed for **2f (**179.1 kJ/mol [56], 172.0 kJ/mol [51]**)** In the Cyt–K^+^ case, the weighted MIA value, 126 kJ/mol, lies between the 110 ± 4 kJ/mol [43] and 135 ± 3 kJ/mol [51] values measured in these laboratories, and is closer to the MIA values computed for **2f** (132.6 kJ/mol [56] and 134 kJ/mol [51]) than those for **2e** (111.3 kJ/mol [56], 116.1 kJ/mol [51]) These considerations imply that a significant (if not dominant) population of the tautomeric forms of Cyt–M^+^ produced by electrospray has MIA values lower than that of **2d**, and the likely candidates for the tautomer population participating in the hydration equilibrium reactions 1 are **2e** and/or **2f**. Because the barriers associated with **2e → 2f** tautomerization, 65.3 kJ/mol [56], 59.9 kJ/mol [51] for Cyt–Na^+^, and 43.2 kJ/mol [51] for Cyt-K^+^, are quite low, such conversion may occur during ion transport through the heated (~380 K) capillary and at the experimental temperatures in the RC.

Similar behavior in the correlation, Figure 4a, obtained for the first and second waters of NAD–Na^+^·(H_2_O)_*n*=1,2_ suggests that both water molecules in these complexes interact directly with the Na^+^ ion. The lower binding energies of the second water molecule in these systems might be accounted for by increasing steric crowding and decreasing effective charge on the Na^+^ ion when the second water molecule is added to the NAD–Na^+^·(H_2_O) complex.

The water binding energies in the sodiated complexes are higher than in the potassiated ones (Tables 1 and 2) This observation is very similar to our previous studies [73–75] and is consistent with the dominating electrostatic interaction between the water molecule and metal ion in the NAB–M^+^·(H_2_O)_*n* = 1,2_ complexes. Because the ionic radius of K^+^ is larger than Na^+^, the charge density should be smaller and, therefore, the electrostatic interaction of H_2_O with NAB–K^+^ is weaker compared with NAB–Na^+^.

## Conclusions

In this study, hydration equilibrium measurements provide information about the water binding energies to sodiated and potassiated nucleic acid bases (uracil, thymine, cytosine and adenine) generated by electrospray ionization. These results, coupled with previously reported tautomeric forms involved in reactant NAB–M^+^ complexes provides insights into their hydrated structures.

The correlation between water binding energies in the NAB–M^+^·(H_2_O) systems and the corresponding metal ion affinity values of nucleobases reveals that in the case of the Cyt–M^+^ species, the population of the tautomeric forms produced by electrospray resembles that formed upon the thermal sublimation of cytosine where the populations of the **2a + 2b** precursors of **2e** and **2f** are predominant. These results indirectly indicate that a substantial amount of canonical cytosine undergoes tautomerization by electrospray ionization, and the thermochemical values obtained for Cyt–M^+^ most likely correspond to a mixture of tautomeric structures.
